# Mammographic screening before age 50 years in the UK: comparison of the radiation risks with the mortality benefits

**DOI:** 10.1038/sj.bjc.6602683

**Published:** 2005-09-05

**Authors:** A Berrington de González, G Reeves

**Affiliations:** 1 Cancer Research UK Epidemiology Unit, University of Oxford, Gibson Building, Radcliffe Infirmary, Oxford OX2 6HE, UK

**Keywords:** mammography, breast cancer, radiation, risk assessment

## Abstract

Mammographic screening before age 50 years is less effective than at older ages and the associated radiation risks are higher. We estimated how many breast cancer deaths could be caused and how many could be prevented by a decade of annual two-view mammographic screening starting at ages 20, 30 and 40 years, respectively, in the UK; for all women, and for women with first-degree relatives affected with breast cancer. We extrapolated from a radiation risk model to estimate the number of radiation-induced breast cancer deaths, and used results from randomised trials, which suggest a reduction in breast cancer mortality of 10–20% in women invited to screening before age 50 years, to estimate the number of deaths that could be prevented. The net change in breast cancer deaths was defined as the number of radiation-induced deaths minus the number of prevented deaths. For all women, assuming a reduction in mortality from screening of 20%, a decade of annual screening was estimated to induce more deaths than it prevents if started at age 20 years and at age 30 years (net increase=0.86 and 0.37 breast cancer deaths, respectively, per 1000 women screened). The corresponding estimate for screening starting at age 40 years was a net decrease of 0.46 deaths/1000 women screened and a zero net change assuming a 10% mortality reduction. Results for women with first-degree relatives with breast cancer were generally in the same direction but, because their background incidence rates are higher, the net increases or decreases were greater. In conclusion, our estimates suggest that a decade of annual two-view mammographic screening before age 40 years would result in a net increase in breast cancer deaths, and that starting at age 40 years could result in a material net decrease only if breast cancer mortality is reduced by about 20% or more in women screened. Although these calculations were based on a number of uncertain parameters, in general, the conclusions were not altered when these parameters were varied within a feasible range.

The National Health Service Breast Screening Programme currently invites women in the UK aged 50–70 years for mammographic screening once every 3 years. Whether screening should be offered to women younger than age 50 years, particularly to those thought to be at a higher than average risk of the disease because of a family history of breast cancer, is a question that is frequently raised. However, mammographic screening before age 50 years is less effective than at older ages possibly because premenopausal women have denser breasts and because the tumours grow more rapidly (Buist *et al*, 2004). Preliminary results from the UK Age Trial suggest that the reduction in breast cancer mortality associated with offering annual mammographic screening from age 40 to 47/48 years may be 10% (Moss *et al*, 2005), which is considerably lower than the 25% reduction achieved in randomised trials offering regular screening to women aged 50–69 years (IARC, 2002).

Exposure to mammographic X-rays confers a risk of radiation-induced breast cancer, which is greater the younger the women are when they are exposed (Preston *et al*, 2002). It is difficult to quantify the risk of radiation-induced breast cancer associated with the low doses of radiation to which women are exposed during mammographic screening using observational studies directly (Ron, 2003). However, the risks can be estimated by extrapolating results from studies of populations exposed to a wider range of radiation doses. In this paper, we estimate the number of radiation-induced breast cancer deaths associated with a decade of annual mammographic screening starting at ages 20, 30 and 40 years using results from a recent pooled analysis of cohort studies of breast cancer risk after radiation exposure (Preston *et al*, 2002). These estimates are then compared with the estimated number of deaths from breast cancer that could be prevented by mammographic screening assuming a 10 or 20% reduction in mortality, respectively, in women screened. Results are presented both for all women and for women who have first-degree relatives affected with breast cancer.

## MATERIALS AND METHODS

### Number of radiation-induced breast cancer deaths

For our calculations, we assumed that before age 50 years women would be screened annually with a two-view mammogram. Data collected on a sample of women in the UK NHS Breast Screening Programme during 2001–2002 and conversion factors for glandular dose give estimates of an average radiation dose to the glandular breast tissue of 4.5 mGy from a two-view mammogram (Dance *et al*, 1999; Young *et al*, 2005); we used this tissue dose and assumed that the dose does not vary with age (Young, 2002).

Estimates of the risk of radiation-induced breast cancer presented here were calculated using an excess relative risk model (ERR), which means that the risk of radiation-induced breast cancer is calculated relative to the estimated ‘underlying’ breast cancer incidence rate for the UK population. The risk model that was used was based on a pooled analysis of three cohort studies from Preston *et al* (2002). Two of these three cohorts were comprised of women with tuberculosis who had multiple fluoroscopy examinations, and hence received very similar types of radiation exposure to those under consideration here (i.e. multiple diagnostic X-rays). The excess relative risk was found to increase linearly with increasing radiation dose and to decrease with increasing attained age. The underlying breast cancer rate that was used was for 2001 and is an estimate of what the current rate would be in the absence of breast cancer screening of women aged 50 years and over (Moss, personal communication). Further details on the model and methods used are provided in the Appendix A.

The estimated risk of radiation-induced breast cancer mortality was then estimated from the risk of radiation-induced breast cancer incidence to enable a direct comparison with the number of deaths that could be prevented by screening. We estimated current age-specific breast cancer survival probabilities by taking the 10-year relative survival probability in England and Wales for women whose breast cancers were diagnosed between 1980 and 1985 (Coleman *et al*, 1999) and scaling it by the ratio of breast cancer mortality rates in 1985 (ONS, 1986) to 2001 (ONS, 2002). This resulted in estimates of current breast cancer survival probabilities of 0.59 for diagnoses between ages 15 and 44 years, 0.65 for diagnoses between ages 45 and 59 years and 0.54 for diagnoses at age 60+ years. The estimated risk of radiation-induced breast cancer incidence at each age was multiplied by 1−the age-specific survival probability to calculate the risk of radiation-induced breast cancer mortality.

Preston *et al* (2002) found that the incidence of breast cancer is increased from about 10 years after initial radiation exposure and remains elevated for at least 50–60 years after exposure. Therefore, the total risk of radiation-induced breast cancer mortality was estimated as a cumulative (lifetime) risk beginning from 10 years after exposure and continuing up to age 85 years, with adjustment for competing causes of death based on UK all cause mortality rates for 2001 (ONS, 2002).

### Number of breast cancer deaths prevented

Results from randomised trials suggest that, at a population level, offering mammographic screening to women aged 50–69 years could reduce breast cancer mortality by 25%, which implies a 35% reduction in mortality among the women who attend regular mammographic screening (IARC, 2002). A review of results from the randomised trials of women aged 40–49 years when they were first offered screening suggests a possible 20% reduction in breast cancer mortality (IARC, 2002). However, this is considered to be the maximum likely reduction as part of this reduction is likely to be due to mammography performed after age 50 years in these trials. Preliminary results (based on surrogate markers) from the UK Age Trial, which was designed specifically to investigate the effect of mammographic screening of women starting at age 40 years compared to starting at age 50 years, suggest that the reduction in breast cancer mortality associated with offering annual mammographic screening from ages 40 to 47/48 years may be 10% (Moss *et al*, 2005). Therefore, the number of deaths that could be prevented by a decade of annual screening for women aged less than 50 years was calculated under two scenarios: assuming, respectively, a 10 or 20% reduction in breast cancer mortality among women screened.

In these calculations, it was necessary to take account of the fact that the mortality reduction would only apply to deaths from cancers that were diagnosed during the screening period. To estimate the proportion of cancer deaths that would have been diagnosed in each screening decade, data on the age at breast cancer death were crossclassified by age at diagnosis for the deaths in England and Wales in 1998 (M Quinn, personal communication). The breast cancer mortality rates that could be reduced by attending screening were then estimated by scaling the UK breast cancer mortality rates for 2001 by the probabilities that the cancer deaths at each age were diagnosed during the screening decade. The number of breast cancer deaths that could be prevented by attending screening was estimated by reducing these scaled rates by 10 and 20%, respectively.

The net change in breast cancer mortality was defined as the number of radiation-induced breast cancer deaths minus the number of deaths that could be prevented per 1000 women screened. A positive net change means that the radiation-induced risks are greater than the mortality benefits and *vice versa*.

Sensitivity analyses were also performed to evaluate the effects of uncertainties in the radiation risk models and mortality benefits on the results.

### Women with a family history of breast cancer

These calculations were repeated for women with first-degree relatives with breast cancer by scaling the estimated underlying UK breast cancer incidence rates and mortality rates by estimates of risk ratios for breast cancer incidence and mortality for women with a family history of breast cancer, compared with women who had no family history of breast cancer. These risk ratios were taken from results of a reanalysis of epidemiological studies of familial breast cancer (Collaborative Group on Hormonal Factors in Breast Cancer, 2001). For example, a woman with one affected first-degree relative was assumed to have an underlying breast cancer incidence rate at ages 45–54 years that was 1.9 times higher than the breast cancer incidence rate in the general population, and for a woman with two affected first-degree relatives the estimated rate for those ages was 3.2 times higher than that in the general population. All other aspects of the calculations were the same as for the general population.

### Years of life lost and gained

Since the radiation-induced cancers are likely to occur later in life, on average, than the deaths that are prevented by screening, we used current age-specific life expectancy for the UK (GAD, 2005) to also estimate the net change in years of life associated with each decade of annual mammographic screening for all women and for women with a family history of breast cancer.

## RESULTS

Table 1 shows the estimated risk of radiation-induced breast cancer mortality associated with a decade of annual screening according to age and family history of breast cancer. A decade of annual mammographic screening starting at age 20 years was estimated to increase breast cancer mortality by 0.91 deaths for every 1000 women screened, which was nearly double the estimate of radiation-induced breast cancer mortality associated with a decade of annual screening starting at age 40 years (0.50 per 1000 women screened, Table 1), and nearly 10 times the estimate for a decade of screening every 3 years starting at age 50 years (0.11 per 1000 women screened). The underlying breast cancer rates are about two to three times higher in women with one or two affected first-degree relatives than in the general population, hence these women were estimated to have cumulative excess risks of radiation-induced breast cancer mortality that were approximately two and three times higher, respectively, than the figures for all women.

**Figure 1:**
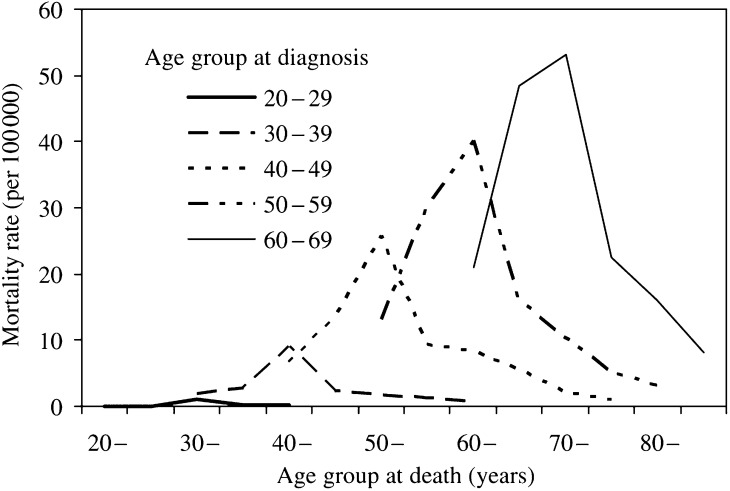
Annual UK age-specific breast cancer mortality rates (per 100 000) according to the age that women were when their breast cancer was diagnosed (20–29 up to 60–69 years).

Figure 1 shows the estimated age-specific breast cancer mortality rates in women according to their age when breast cancer was diagnosed. It can be seen that these mortality rates are higher the older the women are at diagnosis (rates are extremely low for women aged 20–29 years at diagnosis and fairly low at age 30–39 years). It can also be seen that most deaths occur 5–15 years after the diagnosis of breast cancer.

Table 2 shows the estimated reduction in breast cancer mortality associated with a decade of annual screening according to women's age and a family history of breast cancer. A decade of annual mammographic screening was estimated to reduce breast cancer mortality by 0.05 deaths per 1000 women screened starting at age 20 years compared to 0.96 per 1000 women screened starting at age 40 years, assuming a 20% mortality reduction in women screened. Again, because of their higher underlying breast cancer rates, for women with one or two affected first-degree relatives, these figures were at least two and four times higher, respectively.

**Figure 2:**
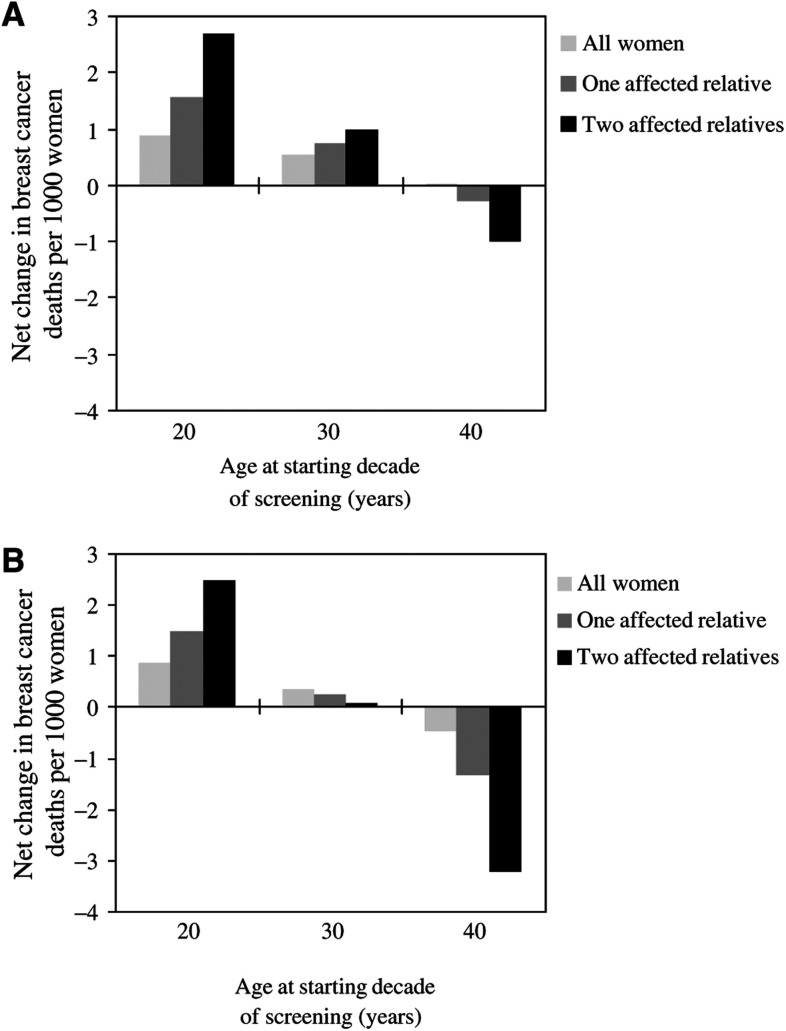
Estimated net change in breast cancer deaths per 1000 women screened in the UK according to age at starting decade of annual screening: all women and women with affected first degree-relatives. (**A**) Assuming a 10% mortality reduction, and (**B**) assuming a 20% mortality reduction.

Figure 2A and B shows the estimated net change in breast cancer mortality associated with a decade of annual screening according to age and a family history of breast cancer for a 10 and 20% reduction in breast cancer mortality, respectively, in women screened. There was a net increase in breast cancer deaths for a decade of annual screening starting at age 20 years or age 30 years, i.e., the number of radiation-induced deaths was greater than the number of deaths prevented, even for women with affected first-degree relatives and regardless of whether a 10 or 20% reduction in mortality was assumed. For a decade of annual screening starting at age 40 years, there was little or no change in the net breast cancer mortality assuming a 10% reduction in breast cancer mortality. However, if a 20% reduction in breast cancer mortality was assumed, there was a net decrease in breast cancer deaths for all women and for women with first degree-relatives with the disease. In this scenario, the greatest net decrease was seen for women with two affected first-degree relatives (Figure 2B).

For all women, a net loss in years of life was estimated for a decade of screening starting at age 20 years, and screening starting at age 30 years was estimated to result in a net gain in years of life if a 20% mortality reduction was assumed (Table 3). For a decade of annual mammographic screening starting at age 40 years, net gains in years of life were estimated for both a 10 and 20% mortality reduction, although the net gain was small for all women (7–20 years per 1000 women screened) compared with the estimated net gain for a decade of screening starting at age 50 years (42 years per 1000 women screened).

We performed a sensitivity analysis to investigate the effect on the estimated net change in breast cancer mortality of varying the assumptions and parameters within a ‘feasible range’ and compared the estimated net change in breast cancer mortality with the estimates from the original parameter values (Table 4). This showed that for a decade of annual screening starting at age 20 years, changing the magnitude of the ERR/Gy by one standard error resulted in the largest % change in the estimated net change in breast cancer mortality, whereas for a decade of annual screening starting at age 40 years, the parameters relating to the percent reduction in mortality associated with screening had the greatest impact. In general, annual mammography before age 40 years did not appear to be beneficial over a wide range of assumptions, but starting screening at age 40 years could be either beneficial or harmful, depending on whether the mortality reduction was assumed to be 20 or 10%.

**Figure 3:**
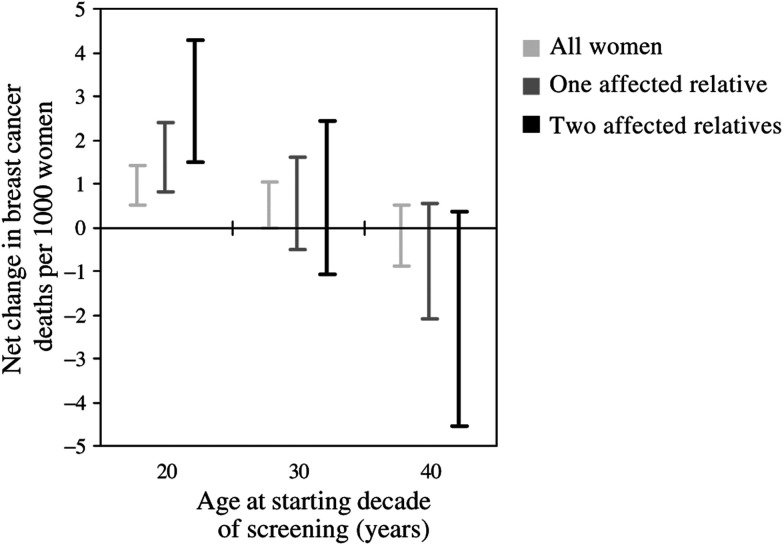
Estimates from the sensitivity analysis of the minimum and maximum net change in breast cancer deaths per 1000 women screened in the UK according to age at starting decade of annual screening: all women and women with affected first-degree relatives.

Varying all assumptions simultaneously within their feasible ranges gives estimates of the possible extreme values of the net changes in breast cancer mortality (Figure 3). In particular, we were interested in whether these extremes suggested potential reversals in the direction of the estimated net change in breast cancer mortality. For a decade of annual screening starting at age 20 years, the minimum estimate of the net change in breast cancer mortality still suggested that more deaths would be induced than prevented for all women and also for those with affected first-degree relatives. The minimum estimates for the net change in breast cancer mortality for a decade of annual screening starting at age 30 years suggested at most small net decreases even with extreme assumptions. For a decade of annual screening starting at age 40 years, there was variation in the net change in breast cancer mortality from small increases to large decreases. For all women, for example, these uncertainties resulted in estimates that ranged from a net increase in breast cancer mortality of 0.50 to a net decrease of 0.88 per 1000 women screened. The ranges of the estimates were widest for women with two affected first-degree relatives, suggesting that the results were most uncertain for this group.

## DISCUSSION

Our estimates suggest that for all women a decade of annual mammographic screening starting at age 20 years would cause more radiation-induced breast cancer deaths than it prevents, and starting at age 30 years it is unlikely to result in a net reduction in breast cancer mortality. However, a decade of annual screening starting at age 40 years could result in a material net decrease in breast cancer mortality if, among women screened, breast cancer mortality is reduced by about 20% or more. If the mortality reduction is 10% in women screened, then a decade of annual screening starting at age 40 years may have little or no net benefit. Results for women with first-degree relatives with breast cancer were generally in the same direction but, because their background incidence rates are higher, the net increases or decreases were greater.

These calculations were necessarily based upon a number of assumptions and parameter estimates; however, the sensitivity analysis suggested that, for the most part, the conclusions were unlikely to be significantly altered by varying the parameters within a feasible range. One exception was for screening starting at age 40 years for all women, where the direction of the net effect was altered by varying the assumptions, however the magnitude of the net effects were modest eitherway.

In this paper, we have focused on the comparison of the radiation-induced breast cancer deaths with the number of breast cancer deaths prevented by screening. However, the radiation-induced cancers are likely to occur later in life, on average, than the deaths that are prevented by screening. Therefore, we also compared the years of life lost and gained by each decade of mammographic screening. The conclusions from these analyses were generally similar to those from the analysis of numbers of deaths, with the exception of estimated small net gains in years of life for a decade of annual screening starting at age 30 years for all women if a 20% mortality reduction from screening was assumed, and also for all women for a decade of screening starting at age 40 years if a 10% mortality reduction was assumed (net gain=2 and 7 years of life per 1000 women screened, respectively).

In our calculations we did not assume that women attend for regular screening after the specific decade of interest, because the question under investigation was the net effect of each decade of screening. The reason for this is that we do not think that screening could be recommended to a certain age group on the basis of guaranteed future screening attendance. Future screening attendance could reduce the magnitude of the risk of radiation-induced breast cancer mortality, if some of these cancers were detected by screening. Therefore, we investigated the effect on the estimated risk of radiation-induced breast cancer mortality of assuming 100% future screening attendance by increasing the breast cancer survival probabilities by 10 or 20% for screening before age 50 years and by 35% after age 50 years. For a 20% mortality reduction due to a decade of annual screening, assuming 100% future screening attendance reduced the net increase in breast cancer deaths from 0.86 to 0.62 per 1000 women screened starting at age 20 years, from 0.37 to 0.16 starting at age 30 years, and for starting at age 40 years this changed the net decrease from 0.46 to 0.64 per 1000 women screened. Therefore, even if we assumed 100% future screening attendance, this would be likely to alter the magnitude of the net change, but not the direction of the result.

In our calculations we assumed women would be screened annually with a two-view mammogram. In the UK Age trial, two-view mammography was used for the first screen only, whereas subsequent rounds used single-view mammography (Moss *et al*, 2005). The radiation dose from a single-view mammogram is 2.5 mGy (Young *et al*, 2005) and so under this screening pattern, the estimated risk of radiation-induced breast cancer mortality for annual mammographic screening from age 40 to 47/48 years would approximately be halved (0.22 breast cancer deaths per 1000 women screened). Although reducing the number of views per screen, or the frequency of screening, will reduce the radiation risk it may also decrease the reduction in breast cancer mortality due to screening.

The estimates of the radiation-induced breast cancer risk were based on the linear no-threshold assumption,that is assuming the radiation risks are linear in dose down to very low doses and that there is no threshold dose below which there is no risk of cancer. A recent review of the available biological and epidemiological evidence concluded that there is direct epidemiological evidence of an excess cancer risk from fractionated radiation doses as low as 50 mGy (Brenner *et al*, 2003), which is approximately the dose received from a decade of annual two-view screening mammograms (45 mGy). Therefore, the assumptions made in this article about the existence of cancer risks at these low dose levels are supported by epidemiological evidence, but the extrapolation is necessary because it is not feasible to quantify the risks using observational studies directly. Brenner *et al* (2003) also reviewed the evidence regarding the most appropriate form of the extrapolation and concluded that the assumption of linearity was most consistent with the experimental evidence and, furthermore, that alternative forms could result in larger as well as smaller risk estimates. As Preston *et al* (2002) found no evidence that fractionated exposure resulted in a lower risk of radiation-induced breast cancer than acute exposure, a dose rate reduction effectiveness factor was not included in these calculations.

The estimates for women with a family history of breast cancer were based upon the assumption that the excess relative risk of radiation-induced breast cancer per unit dose for these women is the same as for all women, that is, that there is no supra or submultiplicative interaction between radiation exposure and a family history of breast cancer. However, BRCA-1 and BRCA-2 mutations appear to be associated with a reduction in efficiency of DNA repair, which suggests that there may be an interaction between these two risk factors, at least for this subgroup of women with a family history of breast cancer (IARC, 2000). To date, only one study has investigated the risk of radiation-induced breast cancer in women with a family history of breast cancer directly, and reported that women with a family history of breast cancer might have a greater relative risk of radiation-induced breast cancer (Ronckers, 2003). Further research into this question is needed, because if this were true then the radiation risks reported in this paper for women with a family history of breast cancer could be underestimates.

The risk model that was used for these calculations was an excess relative risk model based on a pooled analysis of three cohorts, including two cohorts of women who were exposed to multiple fluoroscopy examinations (Preston *et al*, 2002). In their pooled analysis, Preston *et al* found no single excess relative or excess absolute risk model that adequately described the risk of radiation-induced breast cancer across all of the eight cohorts considered. For breast cancer risk estimation in general populations, they suggested the use of their pooled excess absolute risk model, which included four of the possible eight cohort studies. Formal statistical comparison of the fits of the excess relative risk and excess absolute risk models is not possible, but an informal comparison based on deviance values suggested that the excess relative risk model fitted the data marginally better. Furthermore, the assumption underlying the excess absolute risk model is that the risk of radiation-induced breast cancer is not related to the underlying breast cancer incidence rate in the population. This is equivalent to assuming that the relative increase in the risk of breast cancer associated with radiation is actually lower for women with a greater than average baseline risk of breast cancer, such as women with a family history, than it is for other women. As explained above, to date there is little reliable information on the risk of radiation-induced breast cancer specifically in women with a family history of the disease, but we do not think currently that such an assumption is justifiable. However, in the sensitivity analysis, we investigated the effect of using Preston *et al*'s pooled excess absolute risk model for all women, and although the radiation risk estimates were somewhat lower than those estimated using the excess relative risk model, the conclusions were not materially altered (Table 4).

Several previous studies have also estimated the radiation risks associated with mammographic screening of younger women. Feig and Hendrick (1997) estimated the risks from screening women aged 40–49 years and, assuming mortality reductions of between 24 and 36% for screened women, concluded that the radiation risks would be small compared to the mortality benefits. Beemsterboer *et al* (1998) and Mattsson *et al* (2000) focused on the question of whether to start screening all women at age 40 years rather than at age 50 years. Both conclude that this strategy would reduce the net reduction in breast cancer mortality. Mattsson *et al* also concluded that at least a 20% annual reduction in breast cancer mortality was necessary for the reduction in breast cancer mortality to outweigh the radiation risks if screening starts at age 40 years. Finally, Law and Faulkner (2001) considered the question of screening all women younger than age 50 years and those with a family history of breast cancer by estimating the ratio of the number of cancers that might be detected by screening compared to the number of cases induced by radiation from a single mammogram. The interpretation of this ratio is much less straightforward than the comparison of deaths induced to deaths prevented, but the authors suggest that a ratio of 10 : 1 may be necessary to recommend screening and concluded therefore that mammographic screening should certainly not start before age 35 years.

The estimates for women with a family history of breast cancer were for women with one or two affected first-degree relatives, but these estimates could also be applied to other groups of women that have a similarly increased underlying risk of breast cancer. For example, for a woman with one first-degree relative who was diagnosed with breast cancer before age 40 years, the conclusions would be similar to those we have presented for women with two affected first-degree relatives. Furthermore, although our estimates are based on breast cancer incidence and mortality data from the UK, the other parameters that were used in the calculations including the radiation risk models and relative risks for a family history of breast cancer were all based upon data from international studies. Therefore, it is likely that our findings would be broadly applicable to other Western populations with broadly similar breast cancer incidence and mortality rates.

In conclusion, our estimates suggest that a decade of annual mammographic screening before age 40 years would result in a net increase in breast cancer deaths, and that starting at age 40 years could result in a material net decrease in breast cancer deaths if breast cancer mortality is reduced by about 20% or more in women screened. Although these calculations were based on a number of uncertain parameters, in general, the conclusions were not altered when the parameters were varied within a feasible range.

## Figures and Tables

**Table 1:** Estimated cumulative number of radiation-induced breast cancer deaths per 1000 women screened in the UK according to age at starting decade of annual screening: all women and women with affected first-degree relatives

		**Women with affected first-degree relatives**	
**Age at starting decade of screening (years)**	**All women**	**One affected relative**	**Two affected relatives**
20	0.91	1.64	2.80
30	0.72	1.21	1.90
40	0.50	0.79	1.24
			
50^a^	0.11	0.17	0.25
60^a^	0.04	0.06	0.08

a Estimates for screening starting at ages 50 and 60 years are shown for comparison and assume screening every 3 years (current practice in the UK National Health Service Breast Screening Programme).

**Table 2:** Estimated reduction in breast cancer mortality per 1000 women screened in the UK according to age at starting decade of annual screening and assuming a 10 or 20% reduction in breast cancer mortality: all women and women with affected first-degree relatives

			**Women with affected first-degree relatives**	
**Age at starting decade of screening (years)**	**Assumed reduction in breast cancer mortality (%)**	**All women**	**One affected relative**	**Two affected relatives**
20	10	0.02	0.07	0.13
	20	0.05	0.14	0.25
				
30	10	0.18	0.49	0.91
	20	0.35	0.97	1.81
				
40	10	0.48	1.06	2.18
	20	0.96	2.13	4.35
				
50^a^	35	2.27	4.16	7.33
				
60^a^	35	2.53	4.02	6.74

a Estimates for screening starting at ages 50 and 60 years are shown for comparison, and are only presented for a 35% reduction in breast cancer mortality, which is the estimated reduction based on the mammographic screening trials (IARC, 2002).

**Table 3:** Estimated years of life lost due to radiation-induced breast cancer mortality, years of life gained from prevented breast cancer deaths and net change in years of life per 1000 women screened in the UK according to age at starting decade of annual screening: all women and women with affected first-degree relatives

			**Years of life gained per 1000 women screened**		**Net change in years of life per 1000 women screened**	
			**Assumed mortality reduction**		**Assumed mortality reduction**	
**Age at starting decade of screening (years)**		**Years of life lost from radiation- induced breast cancer per 1000 women screened**	**10%**	**20%**	**10%**	**20%**
20	All women	16	1	2	−15	−14
	One affected relative	32	3	7	−28	−25
	Two affected relatives	60	6	12	−54	−48
						
30	All women	10	6	13	−4	2
	One affected relative	18	18	35	0	17
	Two affected relatives	31	33	66	2	35
						
40	All women	5	12	25	7	20
	One affected relative	8	29	57	20	49
	Two affected relatives	14	59	118	45	104
						
50	All women	1	—	43^a^	—	42^a^
	One affected relative	1	—	80^a^	—	79^a^
	Two affected relatives	2	—	144^a^	—	142^a^
						
60	All women	1	—	32^a^	—	31^a^
	One affected relative	0	—	51^a^	—	51^a^
	Two affected relatives	0	—	86^a^	—	86^a^

a Estimates for screening every 3 years starting at ages 50 and 60 years are shown for comparison, and are only presented for a 35% reduction in breast cancer mortality, which is the estimated reduction based on the mammographic screening trials (IARC, 2002).

**Table 4:** Effect of varying the assumed values of certain parameters on the estimated net change in breast cancer deaths per 1000 women screened in the UK according to age at starting decade of annual screening: all women

	**Net change in breast cancer deaths**		
**Description and feasible range**	**Age 20 years**	**Age 30 years**	**Age 40 years**
Baseline scenario – original parameter values^a^	0.86	0.37	−0.46
Mortality reduction of 10%	0.89	0.55	0.02
% cancer deaths that were cancers detected during screening decade (±20%)	0.86–0.88	0.30–0.44	−0.66 to −0.27
Excess absolute risk model for radiation-induced breast cancer	0.73	0.07	−0.76
ERR Gy^−1^ in the radiation risk model (±1 s.e.)	0.67–1.13	0.21–0.58	−0.57 to −0.32
Breast cancer survival (±20%)	0.69–1.05	0.23–0.55	−0.56 to −0.33
Attained age parameter in the radiation risk model (±1 s.e.)	0.83–0.90	0.23–0.41	−0.52 to −0.42

a Assuming a 20% reduction in breast cancer mortality.

## Appendix A

### EXAMPLE OF THE CALCULATIONS FOR A DECADE OF ANNUAL MAMMOGRAPHIC SCREENING STARTING AT AGE 40 YEARS

#### Radiation risk calculations

The model that was used is an excess relative risk model for the risk of radiation-induced breast cancer incidence (*R*_*j*_) at attained age_*j*_, where *λ*_*j*_ is the underlying breast cancer incidence rate in the population of interest at the attained age *j* (Preston *et al*, 2002 – Table 9)

Here, *d*_*k*_ is the radiation dose to the glandular breast tissue from a two-view mammographic screen at age *k*, and the doses are summed up to 10 years prior to attained age *j* to allow for the lag period for the induction of cancer (assumed to be 10 years for breast cancer).

The risk of radiation-induced breast cancer mortality was then calculated by multiplying the risk of radiation-induced breast cancer incidence at each age *j* by 1−the age-specific probability of breast cancer survival (*M*_*j*_). The cumulative risk of radiation-induced breast cancer mortality (CLR) is defined as the sum of these risks from age 50 to 84 years inclusive, adjusted for competing causes of death using all cause survival probabilities (*S*_*j*_).

#### Mortality reduction calculations

Only breast cancer deaths that were from breast cancers diagnosed in women aged 40–49 years could be affected by screening from age 40–49 years. To calculate the age-specific breast cancer mortality rate for cancers that were diagnosed between age 40 and 49 years, the breast cancer mortality rates for women in the general UK population at age *j* (*B*_*j*_) were multiplied by the probability that a breast cancer death at age *j* was diagnosed at ages 40–49 years (*P*_*j*_). If we assume that mammographic screening reduces breast cancer mortality by 20%, then the cumulative lifetime breast cancer mortality prevented by annual mammographic screening from age 40–49 years (*CLP*) is calculated as

For women with a family history of breast cancer, the breast cancer mortality rates are multiplied by estimates of risk ratios (*α*_*j*_) for breast cancer incidence or mortality (*β*_*j*_) for women with a specified family history of breast cancer, either one or two first-degree relatives with breast cancer, compared with women who had no family history of breast cancer. For example, for women with a family history of breast cancer, the *CLP* becomes

#### Excess absolute risk model

In the sensitivity analysis, the effect of assuming an excess absolute risk model was also investigated. This model does not assume that the risk of radiation-induced breast cancer is proportional to the underlying breast cancer rate in the population. In these calculations, the model for the excess risk of radiation-induced breast cancer per Gy where agex is the age at exposure and age is the attained age is also taken from Preston *et al* (2002).
